# Resection planning in extratemporal epilepsy surgery using 3D multimodality imaging and intraoperative MRI

**DOI:** 10.1080/02688697.2016.1265086

**Published:** 2016-12-08

**Authors:** Mark Nowell, Rachel Sparks, Gergely Zombori, Anna Miserocchi, Roman Rodionov, Beate Diehl, Tim Wehner, Mark White, Sebastien Ourselin, Andrew McEvoy, John Duncan

**Affiliations:** ^a^ Department of Clinical and Experimental Epilepsy, UCL Institute of Neurology London UK; ^b^ Epilepsy Society, MRI Unit Chalfont St Peter UK; ^c^ Centre of Medical Imaging and Computing, UCL London UK; ^d^ Department of Neurosurgery, National Hospital for Neurology and Neurosurgery London UK

**Keywords:** Image-guided surgery, neuronavigation, functional neurosurgery, operation

## Abstract

Surgical resection in non-lesional, extratemporal epilepsy, informed by stereoEEG recordings, is challenging. There are no clear borders of resection, and the surgeon is often operating in deep areas of the brain that are difficult to access. We present a technical note where 3D multimodality image integration in EpiNav^TM^ is used to build a planned resection model, based on a previous intracranial EEG evaluation. Intraoperative MRI is then used to ensure a complete resection of the planned model. As stereoEEG becomes more common in the presurgical evaluation of epilepsy, these tools will become increasingly important to facilitate targeted cortical resections.

## The problem

Surgical treatment of nonlesional extratemporal epilepsy is challenging. There is no single diagnostic test for the epileptogenic zone, and patients typically undergo detailed presurgical evaluation comprising of video-EEG telemetry, structural and functional MR imaging and neuropsychometric assessment. Most patients require further advanced imaging such as interictal-ictal single photon emission computed tomography (SPECT) and fluorodeoxyglucose positron emission tomography (FDG-PET), and intracranial electroencephalography (IC-EEG) to further define the site of seizure onset.^1^ StereoEEG (SEEG) is the percutaneous placement of multiple depth electrodes to record the onset and propagation networks of the seizures. As SEEG becomes more common, patients are increasingly identified with epilepsy that originates in deep cortical structures such as the insula and cingulate gyrus. The challenge for the neurosurgeon is to translate the data from the neurophysiology recordings into a 3D space for surgical resection. This is especially important when operating on patients with no structural lesion, where there are no clear anatomical boundaries, and where the epilepsy is thought to originate in deep cortical structures that are difficult to access.

## The solution

EpiNav^TM^ (CMIC, UCL, London, UK) is software used for image integration, advanced visualisation and epilepsy surgery planning.^2^ We describe a pilot case study, where EpiNav^TM^ is used to support the strategic decision making, planning of SEEG and visualisation of implanted depth electrodes. Following recording of seizure activity, advanced segmentation tools are used to highlight electrode contacts of interest, that indicate interictal and early ictal epileptic activity and then to build a planned resection model. This model accounts for the gyral anatomy, blood vessels and the preferred surgical approach, incorporates the electrode contacts implicated in seizure activity and excludes eloquent areas of brain where language and motor function were mapped extra-operatively. This tool facilitates the translation of the neurophysiological data into a 3D surgical plan that can be imported into neuronavigation systems and used for guidance in the operating theatre. Following resection, the patient undergoes intraoperative MRI, and a rigid body coregistration is undertaken to confirm that the resection plan has been followed and that the salient points of interest have been resected.

## Case history

**Figure 1.**
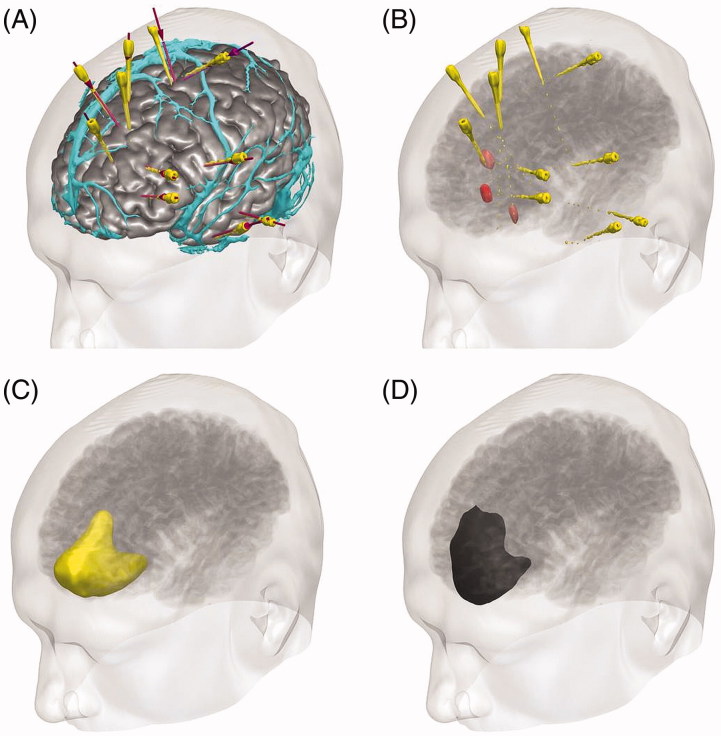
EpiNav^TM^ 3D display of SEEG and resection planning. (A) Cortex model (grey) with overlying veins (cyan), SEEG planned trajectories (violet) and implemented electrodes (yellow). (B) Electrode contacts involved in seizure onset (red) and seizure propagation (pink). (C) Planned resection model incorporating areas of interest (yellow). (D) Completed resection following surgery (black) (see online version for colour figures).

GT is a 30-year old right-handed male, with a 13-year history of medically refractory hyperkinetic seizures with no lateralising signs. His nocturnal seizures occur 2–3 times per week, have no aura and consist of him waking with violent movements of all limbs. His daytime seizures occur 2–3 times per month, and consist of an aura of abnormal feelings in the chest and abdomen followed by violent movements of the limbs and loss of consciousness. Scalp ictal EEG localised to the left frontocentral region. Repeated structural imaging was normal, and FDG-PET showed hypometabolism in the left anteromesial frontal and anterolateral temporal lobes. Magnetoencephalography showed dipole clusters in the left orbitofrontal and left temporal pole. Verbal fluency functional MRI demonstrated left hemisphere language dominance. Neuropyschometry testing showed poor verbal memory, nominal skills and executive skills, consistent with dominant frontal dysfunction. GT underwent an intracranial SEEG study, with electrodes targeting the left orbitofrontal area, inferior and mesial frontal areas, the insula, the cingulate gyrus, the temporal pole, the amygdala and the supplementary motor area (Figure 1(A)). Recordings of his typical seizures suggested seizure onset in the left mesial prefrontal area, with rapid propagation to the anterior cingulum and the medial orbitofrontal region. Language and motor function were mapped in the inferior frontal electrode and the supplementary motor area electrode respectively. GT was offered a cortical resection in the left prefrontal area, extending along the anterior cingulum posteriorly and limited laterally by the superior frontal sulcus.

## Technical considerations

**Figure 2.**
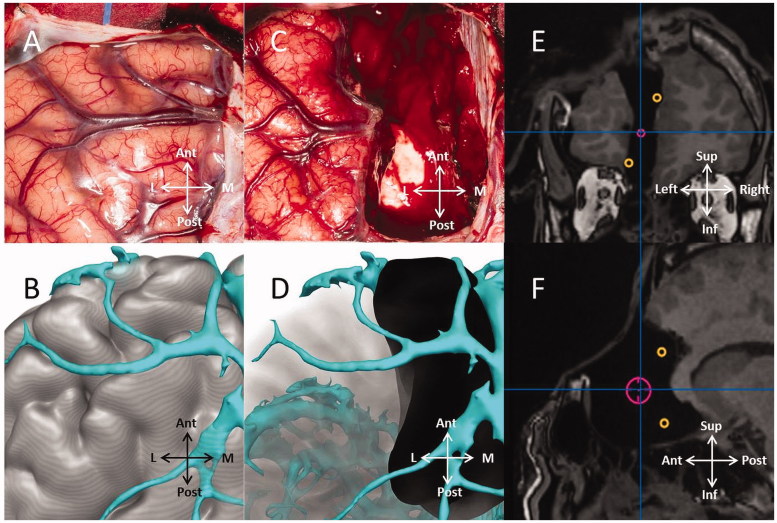
Pre- and post-operative recording of left frontal resection. (A) Intra-operative photograph of left frontal region. (B) EpiNav^TM^ modelling of gyral and vascular anatomy. (C) Intra-operative photograph of left frontal resection. (D) EpiNav^TM^ reconstruction of completed resection. (E and F) Coronal and sagittal intra-operative MRI showing completed resection, incorporating neurophysiological landmarks of ictal onset (red) and propagation to anterior cingulum and medial orbitofrontal area (orange.) (Ant: anterior; Post: posterior; L: lateral; M: medial; Sup: superior; Inf: inferior) (see online version for colour figures).

Image integration, segmentation and 3D visualisation was performed in EpiNav^TM^ (Figure 1). The cortex model was derived from the T1-weighted MRI using a cortical parcellation tool (Freesurfer, Version 5.0.0, Martinos Centre for Biomedical Imaging, Charlestown, MA). The model of cortical veins was derived from 3D phase contrast MRI and T1 weighted-MRI with gadolinium enhancement. The positions of the implanted electrodes were derived from the post-implantation CT. The contacts implicated in seizure origin and propagation were manually segmented and dilated, to build surface landmarks for resection. Using an advanced segmentation tool within EpiNav^TM^, a planned resection was generated in the form of a 3D model, that incorporated these landmarks and that was limited by the gyral and vascular anatomy. Models were exported as binary labelled masks in nifti format on a neuronavigation T1-weighted image. They were imported into iPlan (BrainLab, Feldkirchen, Germany), and coregistered to the neuronavigation scan undertaken at the beginning of the operation in the interventional MR suite. The resection model and landmarks provided guidance for the neurosurgeon during the resection procedure. At the end of the operation, the extent of the proposed resection was confirmed with an intraoperative MRI that was coregistered to the previous MRI with the models overlaid (Figure 2).

## Future work/challenges

We report the combined use of 3D multimodality imaging and intraoperative MRI to guide the surgical management of nonlesional and extratemporal epilepsy. The generation of a planned resection area clarifies the surgical strategy between the surgeon and neurophysiologist and the use of intraoperative MRI gives the surgeon confidence that the proposed resection is complete. These tools are increasingly needed as more challenging nonlesional, extratemporal surgery takes place, informed by SEEG recordings.^3^ We are now refining the segmentation tools in EpiNav^TM^, tailoring them to the needs of visualization of electrode contacts and to epilepsy surgery. We will also incorporate tractographic representations of important transiting white matter pathways, such as the uncinate fasciculus, inferior fronto-occipital fasciculus and arcuate fasciculus, to further aid planning and improve the safety of surgery in these deep cortical areas.
